# Postthrombotic Syndrome: Surgical Possibilities

**DOI:** 10.1155/2012/520604

**Published:** 2011-10-31

**Authors:** Ajay K. Khanna, Shivanshu Singh

**Affiliations:** Department of General Surgery, Institute of Medical Sciences, Banaras Hindu University, Varanasi 221005, India

## Abstract

Postthrombotic syndrome (PTS) is a late outcome of deep vein thrombosis characterized by cramping pain, swelling, hyperpigmentation, eczema, lipodermatosclerosis, and ulceration in the leg due to increased venous outflow resistance and reflux venous flow. Newer surgical and endovascular interventions have a promising result in the management of postthrombotic syndrome. Early surgical or endovascular interventions in appropriately selected patients may decrease the incidence of recurrent ulceration and skin changes and provide a better quality of life. Duplex and IVUS (intravenous ultrasound) along with venography serve as cornerstone investigative tools for assessment of reflux and obstruction. Venous obstruction, if present, should be addressed earlier than reflux. It requires endovenous stenting, endophlebectomy, or open bypass procedures. Venous stripping, foam sclerotherapy, radiofrequency, or laser ablation are used to abolish superficial venous reflux. Valvuloplasty procedures are useful for incompetent but intact deep venous valves, while transposition or axillary vein autotransplantation is done for completely destroyed valves.

## 1. Introduction

Postthrombotic syndrome (PTS) affects nearly 23–60% of patients following an episode of deep vein thrombosis (DVT) [1]. Incidence of venous ulcers is around 3–5% [2]. Development of PTS is the principal determinant of health-related quality of life after DVT [3]. PTS is associated with worse physical quality of life and increased pain [4]. The annual health care cost of PTS in the United States is approximately $200 million [5]. The pathophysiology of PTS involves two key processes, namely, venous outflow obstruction as a result of partial recanalization of thrombus with decreased compliance of thrombotic vein and reflux venous flow due to valvular incompetence. Valve station fibrosis leads to luminal narrowing and valve redundancy or widening of commissural valve angle. These components result in distal venous stasis and venous hypertension in the lower limb. Reflux occurs not only in segments distal to postthrombotic stenosis but also in areas remote from them [6].

Work-up includes meticulous clinical history and examination. Duplex sonography of affected limb has become the initial investigation of choice. It can qualitatively identify site of reflux and stenosis. Presence of echoic lumen, reduced compressibility, impaired augmentation of flow on distal compression, and reduced or absent phasity is qualitative parameters to identify venous thrombus [7]. Quantitative assessment of overall severity of reflux when multiple segments are involved can be studied. At present, multisegment score or presence of axial reflux are the best available measures [8]. Valve closure time has failed to have any clinical utility [9]. One of the most important drawbacks of duplex is its inability to properly assess iliac vein thrombus. Intravascular ultrasound (IVUS ) provides better images of iliac vein and inferior vena cava thrombi [10]. It is readily used during stent procedures. Ascending venography is an invasive method of getting a panoramic view of lower limb venous outflow in infrainguinal area. Efficient collateralization is seen in femoropopliteal postthrombotic stenosis via profunda femoris vein. Problem of distal venous hypertension occurs in iliac vein stenosis due to poor collateral formation in the pelvic veins. This is the reason why iliocaval postthrombotic stenosis needs intervention to reduce the impact of postthrombotic syndrome. Ambulatory venous pressure (AVP) provides an overall assessment of venous dysfunction [11]. AVP is maximum in limbs having venous obstruction with reflux than having either of the two alone. Investigations like magnetic resonance imaging and computed tomography provide three-dimensional view of the venous tree.

Compression therapy in the form of elastic stockings, unna boots, have been the traditional method of managing such patients. High level of noncompliance is a major limiting factor resulting in their failure [12]. Newer devices like Veinoplus have been tried to provide electrical stimulation to muscle calf pump. Contractions of calf muscle compress deep veins and improve the blood flow against resistance, decrease amount of reflux and the amount of venous stasis [13–15]. These all should be considered complimentary to newer surgical and endovascular techniques. The type of surgical or endovascular intervention depends upon the site and type of pathology. Venous obstruction, if present, should be treated earlier than reflux [3] with iliofemoral stenting, endophlebectomy, or venous bypass procedures. Venous bypass procedures include crossover bypass or inline bypass procedure. Superficial venous reflux is managed earlier than deep reflux with stripping, liquid compression, or foam sclerotherapy; endovenous radiofrequency or laser ablation of saphenous vein. Deep vein valve repair is considered a second-stage operation when minimally invasive therapies have failed [3]. Though superficial reflux is managed earlier than deep reflux, it has been suggested that both may be managed concomitantly [3]. Use of bioprosthetic valves (e.g., Portland valve), cryopreserved valves, gracilis sling procedure has been largely discouraging [3]. The modified Italian neovalve reconstruction seemed to improve valve continence result significantly [16]. Still, strict guidelines or criteria regarding who will be benefited most are not established [17]. Patients who are considered for surgical or endovascular interventions include those who had (1) failure of conservative therapy, (2) recurrent complications of postthrombotic syndrome, and (3) younger population suffering with this syndrome. Stasis skin changes are often the main indications of surgery [2].

## 2. Interventions for Iliofemorocaval Obstruction

### 2.1. Iliocaval Stenting

It has become the “procedure of choice” for the management of iliofemorocaval obstruction [18]. Compared to bypass procedures it is relatively simple, has lower risk, it's an OPD basis procedure, has excellent stent patency and better symptom improvement. Under ultrasound guidance, ipsilateral femoral vein is accessed. Iliocaval thrombus is visualized by IVUS. The stenotic lesions are dilated by 15-16 mm balloon followed by placement of self-expanding stent. Intraoperative anticoagulation is done with minimal dose of heparin followed by daily dosage with aspirin. Gianturco Z stent has been used for stenting wide calibre vessels like inferior vena cava. Wallstent has been used for inferior vena cava, pelvic veins, and larger veins of thigh region. For medium-sized vessels, metallic stents with smaller profile, self-expanding, and longitudinal flexibility are used. Commonly used diameters are 12–14 mm. Hartung et al. found a technical success rate of stenting procedure to be 95.5% [19]. Venous clinical severity score had a median improvement from 8.5 to 2, and median venous disability score improved from 2 to 0. Cumulative primary, assisted primary, and secondary patency rates of the venous segments at 3 years were 73%, 88%, and 90%, respectively, in intention to treat. The survival rate was 100% at 1 year and 97.3% at 5 years [19].

### 2.2. Crossover Bypass Procedure (Palma-Dale Procedure [20])

These procedures are done in cases of unsuccessful stenting attempts, stent failure, and long occlusions where stenting may not be feasible. They are more invasive, risky interventions and require longer anticoagulation therapy. According to Vollmar [21], prerequisites for success of cross-femoral grafts are patent contralateral iliofemoral vein and caval run-off; a supine resting pressure gradient in excess of 4 to 5 mmHg between the femoral veins in the involved and contralateral limbs; adequate distal venous system (a patent profunda femoris vein, preferably with an open or partially recanalized superficial femoral vein ); a patent and competent greater saphenous vein on the recipient [run-off] side with a minimal diameter of 4 to 5 mm and no varicosities. In the crossover bypass, the great saphenous vein of the nonaffected limb (donor limb) is exposed and rotated at the saphenofemoral junction. An alternative to the use of autologous saphenous vein is use of a 10 mm PTFE (polytetrafluroethylene) graft for femoro-femoral bypass procedure when adequate calibre saphenous vein is not available. The PTFE graft is more thrombogenic than the autologous graft. Under general anaesthesia, vertical incisions are made in both the groin regions to expose the common femoral, proximal superficial femoral, profunda femoris vein and other femoral tributaries. The saphenous vein graft from nonaffected limb is prepared. A deep tunnel in subcutaneous plane is made in suprapubic and subsartorial region. The saphenous vein is passed through these tunnels towards the affected side taking great care to avoid twisting of the vessel. It is anastomosed in an end to side fashion to the common femoral or proximal popliteal vein distal to the site of iliofemoral obstruction. An arteriovenous fistula is made between posterior tibial artery and vein to increase flow through the graft thus maintaining graft patency [22]. The fistula is usually closed 4 to 8 weeks after surgery. Anticoagulation therapy is given for at least 45 days in the postoperative period. In a study on 78 limbs, Husni reported a “clinical success” rate of 74% and patency was 73% during 7 to 144 months of followup [23]. Halliday et al. reported a cumulative patency rate of 75% during 5 yrs of followup [24].

### 2.3. In-Line Bypass Procedure

This procedure may be performed in the femoroiliocaval obstruction associated with segmental obstruction. Expanded PTFE graft (ePTFE graft) is most commonly used. An arteriovenous fistula is created distally to maintain adequate inflow. Life-long anticoagulation is usually required. Jost et al. showed a secondary patency rate of 54% at 2 years [25].

### 2.4. Endophlebectomy

It is an open surgical technique in which the postthrombotic vein is longitudinally exposed at various segments and the synechiae attached to the intimal layer are carefully removed with scissors at the base. Removal of the constricting bands increases the inflow in iliocaval stenting and outflow for vein valve transposition or transfer and increase calf outflow. Puggioni and Lurie [26] reported a case series of 13 patients in which surgical disobliteration was performed in 23 deep venous segments with 14 deep venous reconstructions. In 77% of patients, the treated segments remained primarily patent at mean followup of 11 months. Overall, secondary patency rate was 93%.

## 3. Interventions to Correct Venous Reflux

Venous reflux in PTS can occur at three sites, namely, superficial venous system, deep, or at perforator level. The superficial reflux being easier to manage is attended earlier than more complicated deep venous reflux. Even in the presence of concomitant deep venous reflux, superficial venous surgery has shown to abolish deep venous reflux in 50% of such limbs and a 77% ulcer healing rate at 12 months [27]. Surgery for deep venous reflux includes valvuloplasty for intact but incompetent valves, valve transposition, or axillary vein autotransplantation for destroyed valves. The first open valvuloplasty was performed by Dr. Kistner in 1968 [28].

### 3.1. Ablation of Superficial Venous Reflux

Saphenous vein stripping is still the standard in managing truncal reflux in saphenous vein. Liquid or foam compression sclerotherapy, endovascular radiofrequency, or laser ablation of the great saphenous vein have all been used and have been rewarding. Liquid compression sclerotherapy is effective for cases of superficial venectasias and nontruncal varicosities with competent great and small saphenous vein [29]. Clinical and hemodynamic follow-up results have shown that foam sclerotherapy is better than liquid compression sclerotherapy for truncal reflux in great saphenous vein [30, 31]. Trials comparing stripping with endovascular radiofrequency ablation have shown lesser pain, shorter hospital stay, and earlier return to work in radiofrequency ablation group. However, at the end of 4 months, no significant difference was found in the two groups [32, 33]. Neglen and associates have combined iliofemoral stenting with obliteration of superficial venous reflux and found that it is safe, effective and minimally invasive single-stage procedure [34]. Ablation of superficial venous reflux along with subcutaneous fasciotomy for chronic and recurrent venous ulcers improves ulcer healing or success of skin grafting [35].

### 3.2. Interventions for Perforator Incompetence

Role of surgical treatment for perforator incompetence in the management of PTS has not been studied well. This is because incompetent perforators are rarely found in isolation in cases of postthrombotic syndrome [36]. The incompetent perforators are treated by SEPS (subfascial endoscopic perforator surgery) or ultrasound guided therapeutic radiofrequency ablation.

### 3.3. Interventions for Deep Venous Reflux

The first and second proximal femoral valve stations are surgically approached via an ipsilateral groin incision made in the direction of vessels. Profunda femoris exposure, if required, needs dissection of sartorius muscle fascia. By performing positive and negative strip tests, reflux at the valve station is assessed. Reflux is demonstrated by venous refilling distally. The adventitia at the particular valve station is carefully dissected to expose the valve attachment lines. Failure to identify the attachment lines implies that the particular valve is completely destroyed, thus not suitable for valvuloplasty. Either transposition or autologous vein transfer may be required in such cases [37].

#### 3.3.1. Internal Valvuloplasty

Following venotomy, the redundant valve cusps are exposed and apposed to the vein wall under direct vision with the help of 7–0 polypropylene suture. Plication of approximately 20% of valve length is usually sufficient to prevent reflux [38]. Dr. Kistner used longitudinal venotomy approach and opened the valve through anterior commissure [39]. Other approaches are supravalvular transverse venotomy without opening the valve [40], T-shaped venotomy [41]. A novel technique of trapdoor internal valvuloplasty has been described by Dr. Tripathi and Ktenidis [42]. After commissure identification, transverse venotomy involving half the circumference is made from the axis of the centre of one commissure to the other, one centimetre above and below the target valve. With the help of specially designed angled retractors, the valve cusps are kept away from the commissural axis. The anterior ends of the two transverse venotomy incisions are connected by a longitudinal venotomy along the more anterior of the two commissures. This creates a trapdoor at the level of target valve. It has the benefits of being technically less demanding and anatomically better defined and physiologically acceptable. The basic aim of all techniques is one, that is, plication of the redundant valve.

#### 3.3.2. External Valvuloplasty

This technique involves transmural suturing through the valve attachment lines without performing a venotomy. It results in narrowing of the commissural angles, thus making the valves competent. Several modifications have been introduced since long-term results have shown it to be less durable repair. Limited anterior plication involves continuous mattress suturing from a point 3 to 4 mm proximal to the valve cusp insertion lines up to the angle of valve cusp insertion [43]. The aim was to reduce the amount of vein dissection and reducing the risk of progressive venous dilatation which occurs in some cases of valvuloplasty. It has promising long-term follow-up results in terms of improved venous refilling and decreased ambulatory venous pressure [43]. In 1991, Gloviczki and colleagues [44] described femoral vein valve repair under angioscopic guidance without performing a venotomy. The angioscope is introduced through the saphenous vein or one of its tributaries and navigated under vision through the common femoral vein to the bifurcation of femoral vein. If found to have primary valvular incompetence, repair of valve is done by external placement of 7–0 polypropylene sutures. Suture is placed lateral to the site of insertion of each valve. The elongated valves are gradually shortened. Valve competency is checked by infusion of irrigation fluid via the angioscope. An external PTFE cuff of size 1 to 1.5 cm width is placed around the site of repair to prevent venous dilatation.

#### 3.3.3. Transcommissural Valvuloplasty

This technique offers the advantage of avoiding venotomy as well as need of an angioscope. Performed in cases of intact but incompetent valves, it is safe, simple, rapid, and multiple valve stations can be repaired at the same setting. An inverted V-shape is formed by the apices of the cusps of bicuspid venous valve. After adventitial dissection and identification of valve station, a through and through transluminal resuspension suture is passed obliquely across the inverted “V”, traversing both the cusps near their attachment to the wall. Involvement of both the valve cusps is evident by puckering of the valve attachment lines. Further two to four more interrupted stitches are placed distally, each of them being less deep and less oblique. The last stitch is at one to two mm beyond the point of maximum bulge at the valve station. The aim is to shorten valve cusp length at each end of commissure by nearly 20%. Similar procedure is repeated at the other end of the cusp. Strip test is performed cautiously to check for valve competence. The internal valvuloplasty corrects the valve cusps only without considering the commissural angles while the external valvuloplasty only corrects the commissural angle without considering the valve cusps. The advantage of transcommissural valvuloplasty is that it corrects both the redundant valve cusps as well as commissural angles. The cumulative competency rates of 140 sites were 84% at 12 months, 72% at 24 months, and 59% at 30 months [28]. Median time to failure was 11 to 16 months (range 2–35 months) depending on the criteria used [28]. Among 36 multiple valves repaired, duplex scan revealed 11 failures. All 11 valves were single failures (i.e., only one of the repaired valves in the individual limb failed, with the other(s) remaining competent) [28]. The cumulative competency rate of the transcommissural repair is 59% to 69% depending on reflux criteria used. It is comparable to those reported for internal and external repair [45–47].

#### 3.3.4. Neovalve Reconstruction

 The Italian neovalve reconstruction is a technique of creating an antireflux mechanism. In this technique, the femoral vein is exposed via a 10 cm long longitudinal incision on the lateral border of sartorius extending up to 10–15 cm below the inguinal ligament. Intraoperative identification of incompetent valve station is most crucial though duplex and other preoperative tests may be used. A longitudinal or transverse venotomy of 2-3 cm length is performed. With the help of ophthalmic blade or microscissors, the intimal dissection is performed to raise an intimal flap. The depth of dissection depends on the vein wall thickness. A monocuspid or a bicuspid valve can be created in this fashion. The size of flap is properly assessed to prevent valve prolapse in case of excessive width. In a variation to the original technique, Lugli and associates stitched the free edge of the flap to the vein wall by 7/0 suture [16]. This prevents reattachment of the flap to the original vein wall that had resulted in failure of neovalve within a short period after surgery in earlier series. Contraindications to neovalve reconstruction include severely limited ambulation, thrombophilia, bleeding diathesis, severe comorbidity, possibility of other standard techniques like femoral transposition, or valve transplant [16]. Cumulative ulcer healing was 7.7/100 patient-months (16 cases per 206 patient-months) in the first series and 30.7/100 patient-months (20 cases per 65 patient-months) in the second [16]. The cumulative patency rates were 16 cases per 919 patient-months (1.7/100 patient-months) and 21 per 228 patient-months (9.2/100 patient-months), respectively, in the first and second series [16]. Postoperative duplex scan and air plethysmography measurements showed a significant improvement (*P* < 0.001) [48]. Thus, neovalve reconstruction seemed to be effective in restoring femoral competence in postthrombotic reflux.

#### 3.3.5. Prosthetic Venous Valves

The need for prosthetic venous valves arises in cases of end-state chronic deep venous insufficiency afflicted with recurrent or resistant venous ulceration in which the available medical therapy and interventional procedures like superficial venous ablation or perforator surgery have failed to provide any clinical or hemodynamic benefit. Majority of the attempts of prosthetic venous valve repair have not shown good results. A bioprosthetic Portland valve (bicuspid square stent -based venous valve made from small intestinal submucosa) has been studied after percutaneous placement in the sheep external jugular vein [49]. It has 88% patency and competency rate. Valve tilting led to occlusion and valve insufficiency in three experimental animals [49]. Variations have been made to prevent tilting and to provide a longer cusp which is supposed to be more hemodynamic structure. Valve thickening is another problem with the prosthetic valves. Cryopreserved valves are superficial femoral vein containing valve allograft made by Cryo Life, Inc, Kennesaw, Georgia. They can prevent venous reflux up to 125 mmHg of retrograde venous pressure. They may require valvuloplasty at the time of implantation for optimal performance and competency [50]. The cryopreserved valve allografts have failed in early and midterm clinical trial and are not considered suitable for treating DVT [51]. Graft rejection is a concerning issue regarding graft failure. T-cell immunosuppressive drugs like cyclosporine and azathioprine may prevent graft rejection. Another technique of restoring deep venous valve competence involves intussusception of the vein into itself [52]. The bicuspid valve is made by two sutures placed at 180 degree to hold the inner vein wall in correct position. Short-term patency was good with competency rate of 90–100%. But hemodynamic results are not as fair as in the case of native valves. Modifications like thinning of the adventitia and partly of the media to decrease the thickness of these valves have been done and studied in canine model [53]. Problem of valve thrombosis is a limiting factor in prosthetic valve as compared to native valves. Repopulating a decellularized external vein containing valve allograft with donor smooth muscle cells and endothelial cells has been attempted to make the transplant quite similar to an autogenous vein in sheep experiments [54]. 75% of the valves (nine out of twelve) were patent and competent at 12 weeks. Two were affected by the neointimal growth and one had occluded [54]. Use of Z-type stent configuration with metal exoskeleton and a vein containing the valve lining inside has been used. The Z-configuration allows expansion in the area of valve which is considered essential for proper valve cleansing and long-term function [55].

#### 3.3.6. Axillary Vein Autotransplantation

First described in humans by Taheri in 1982 [56], this procedure is done in cases of deep venous reflux with destroyed valves. A 2-3 cm of axillary vein segment containing a competent valve or a reparable one is removed. The femoral vein is accessed, and the segment containing the incompetent valve is removed. Presence of intraluminal synechiae in the femoral vein near the site of anastomosis requires excision to create a sizeable lumen for proper anastomosis [57]. If the axillary vein valve explant is incompetent (as seen in 40% of the axillary vein explants), a bench repair by transcommissural external valvuloplasty technique is done before anastomosis [37, 38]. With the help of interrupted sutures, the proximal anastomosis of femoral vein with the axillary vein segment is performed. Proximal anastomosis is done earlier than the distal anastomosis as this allows to check for valvular competence and causes distension and lengthening of the axillary vein segment, thus facilitating better distal anastomosis. An external PTFE sleeve is placed over the axillary vein graft and secured in position by adventitial stitches. It prevents future dilatation of the graft segment.

#### 3.3.7. Valve Transposition

It is a technique in which the incompetent venous system with destroyed valves is placed distal to the competent valve. Prerequisite for this procedure is that at least a single axial venous valve in the groin area is competent. Examples include transaction of incompetent femoral vein and reimplantation distal to the competent valve in the profunda femoris vein or distal to the competent great saphenous vein. Similarly, the incompetent profunda femoris vein can be transposed distal to the competent femoral vein valve. From the results of using the ipsilateral great saphenous vein transposition, it has been found to be safe and effective with good mid-term results, especially for pain. For ulcers, the primary success rate was 55% but it increased to 84% with proper surveillance and treatment of secondary insufficiency of the superficial venous system [58].

## 4. Conclusion

Postthrombotic syndrome is a challenging complication of deep vein thrombosis requiring multimodal approach for appropriate management. Prevention is better than cure holds true in this case. Identification of patients at risk for deep vein thrombosis is essential. Prevention of DVT in these high groups by using mechanical and chemoprophylaxis agents is required to decrease the incidence and complications of postthrombotic syndrome. Though many surgical options have been described, they are performed only at selected centres and are not full proof. The first line and easily available management includes use of graduated compress ion stockings, pneumatic compression devices, oral and injectable anticoagulants. Surgery or endovascular therapy, if feasible, should be considered complementary to medical management. Venous outflow obstruction is treated earlier than venous reflux. Superficial venous reflux is managed before treatment of deep venous reflux. Surgical management is a more definitive treatment than the conservative treatment.
